# Inhibiting Hedgehog: New Insights into a Developmentally Important Signaling Pathway

**DOI:** 10.1371/journal.pbio.0040258

**Published:** 2006-07-18

**Authors:** Mary Hoff

What transforms a ball of undifferentiated cells into an organism with a nervous system, digestive tract, and other specialized body parts? Among the proteins that play an important role is one with the unlikely name of hedgehog (Hh). When Hh attaches to a transmembrane protein known as patched (Ptch1), it initiates a series of molecular interactions that lead to activation of the transcription factor Gli (for “glioma associated”) and the onset of key events in embryonic differentiation.

We know this process involves freeing a second transmembrane protein, smoothened (Smo), from inhibition. But how do Hh and Ptch1 accomplish this? Scientists would like to know because the ability of this signaling pathway to function properly makes the difference between normal development and devastating abnormalities—and because the pathway is also implicated in tumor growth.

Maarten F. Bijlsma, Maikel P. Peppelenbosch, and colleagues began their attempt to find out by noting that previous studies show that enzymes used to make cholesterol are involved in the pathway; that Ptch1 and Smo don’t necessarily bond to each other; that Ptch1 looks like other proteins that pump molecules from one side of the cell membrane to the other; and that cholesterol-like molecules can inhibit the pathway. Based on that information, the researchers hypothesized that when—and only when—Ptch1 is unencumbered by Hh, it pumps a cholesterol-like molecule into the extracellular space, where it inhibits Smo.

To test this, the researchers developed an experimental system made up of fibroblasts modified to luminesce when Gli is active (called reporter cells), and to overexpress various combinations of Ptch1, Smo, and Gli. When they mixed reporter cells overexpressing Smo with cells overexpressing Ptch1, Gli activation in the reporter cells was reduced. When they mixed them instead with cells in which Ptch1-producing genes were silenced, Gli activation increased. After performing additional tests to eliminate alternative explanations, the team concluded that Ptch1 inhibits Smo through an intermediary, and that the intermediary molecule can exert its influence between individual cells.

The researchers next exposed reporter cells to a medium that had contained Ptch1-overexpressing cells, and found Gli activation to be strongly inhibited. However, when they exposed reporter cells to a serum-free, Ptch-conditioned medium, they found no inhibition. Since serum-free medium doesn’t contain lipoproteins, they concluded that a lipoprotein is involved. Further tests suggested that the lipoprotein acts by helping transport a 3β-hydroxysteroid involved in the pathway inhibition.

Interestingly, the researchers noted that certain people with Hh signaling problems have elevated levels of a particular hydroxysteroid, 7-dehydrocholesterol (7-DHC). This led them to test the link between 7-DHC and Gli activity in mouse cells, which in turn led to the conclusion that 7-DHC indeed participates in Ptch1’s inhibition of Smo.

But is the Smo-inhibiting molecule actually 7-DHC or a compound derived from 7-DHC? When the researchers exposed medium from 7-DHC-producing mouse cells to ultraviolet radiation—which changes 7-DHC into vitamin D3—or used vitamin D3 in place of 7-DHC, Hh pathway inhibition was even stronger. They also showed that vitamin D3 binds to Smo, and that it inhibits the Hh pathway in live zebrafish embryos.

Putting it all together, the researchers concluded that when Hh isn’t present, Ptch1 pumps (pro)-vitamin D3 (i.e., either 7-DHC or vitamin D3) into the extracellular space, where the hydroxysteroid grabs onto Smo, inhibiting Gli activation. When Hh binds to Ptch1, the pump grinds to a halt, Smo is freed of inhibition, and transcription of developmentally important genes kicks in.

This new knowledge of the Hh signaling pathway shows how some cells can affect neighboring cells’ development, and it helps explain some of the problems associated with mutations affecting cholesterol biosynthesis. Because the Hh pathway is linked with certain cancers, it also has implications for tumor development. Additional work is now underway to see whether this new understanding of Ptch1’s intercellular inhibition of Smo can be applied to help suppress tumor growth.

## 

**Figure:**
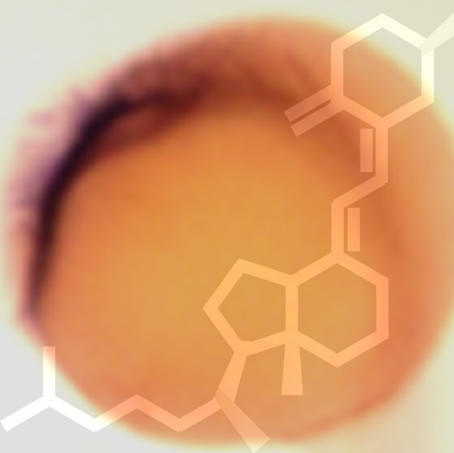
Structure of the vitamin D3 molecule overlaying a photograph of a zebrafish embryo stained for hedgehog pathway activity.

